# Exploring health and wellness with First Nations communities at the “Knowing Your Health Symposium”

**DOI:** 10.1177/08404704221084042

**Published:** 2022-05-25

**Authors:** John Bosco Acharibasam, Meghan Chapados, Jennifer Langan, Danette Starblanket, Mikayla Hagel

**Affiliations:** 1 12371 University of Saskatchewan, Saskatoon, Saskatchewan, Canada.; 2 12371 University of Regina, Regina, Saskatchewan, Canada.

## Abstract

Indigenous older adults living in rural communities require accessibility to and readiness for new technologies to support the monitoring of health data and health status, as well as dementia education. Morning Star Lodge partnered with the File Hills Qu'Appelle Tribal Council, a Community Research Advisory Committee and All Nations Hope Network to bring a diverse group of First Nations community members to the “Knowing Your Health Symposium” to learn about traditional health and First Nations’ wellness. Indigenous research methods and community-based involvement informed and guided the research. An environmental scan was conducted relating to co-researchers’ nutrition, exercise, and self-management of health and health issues through an anonymous survey distributed at the symposium. The purpose of the symposium was to provide communities with information about healthy lifestyles as it relates to dementia and equip community members with the ability to make constructive decisions regarding their health.

## Introduction

Accessibility to culturally appropriate technology for Indigenous older adults is a major issue in ageing research.^1,2^ Indigenous older adults living in rural communities have multiple morbidities that may lead to Early Onset Dementia (EOD) yet lack the accessibility to and readiness for new technologies to monitor health data and consequently health status. Compounding this problem is the fact that Indigenous older adults living in rural communities also lack the health education needed to understand the ways nutrition, exercise, and self-management of health issues can impact multiple morbidities and how multiple morbidities can be related to early dementia onset.

Although earlier studies^3,4^ in Canada have examined the relationship between multimorbidities and EOD among older adults, several gaps in research remain. The previous studies were among the general population and did not specifically examine how the combination of technology and health education can impact EOD among Indigenous populations. By focusing on Indigenous populations, the research aims to improve access to culturally appropriate technology for Indigenous older adults and to enhance healthy ageing in place.

Morning Star Lodge partnered with the File Hills Qu'Appelle Tribal Council (FHQTC), a Community Research Advisory Committee (CRAC) and All Nations Hope Network (ANHN) to bring a diverse group of First Nations community members to the “Knowing Your Health Symposium” to learn about traditional health and to engage in a constructive dialogue regarding First Nations’ wellness. Our objectives were to engage in dialogue on Indigenous nutrition, exercise, and self-management of health and the relationship to cognitive function and dementia and gather baseline data on the health behaviour, specifically nutrition, exercise, and self-management of health among Indigenous peoples.

The symposium was hosted during the Treaty 4 Gathering (September 14, 2019), a major event that brought in hundreds of people for a week of planned celebrations, which included 11 First Nations communities who comprise the FHQTC. During the symposium the research team distributed a questionnaire to examine the relationship between nutrition, exercise, lifestyle, and the prevention of dementia. In addition, partners such as FHQTC, ANHN, All Nations Healing Hospital (ANHH), Elders and Knowledge Keepers, Chiefs and other community leaders, all provided keynote presentations related to dementia and community health.

At the symposium, Morning Star Lodge also invited FHQTC, ANHN, CRAC members, ANHH, and dieticians to host information tables at the symposium, where co-researchers engaged in and learned about health information and had opportunities to get their glucose and blood pressure levels tested as part of the “Knowing Your Health” initiative. In addition, an Indigenous Chef was hired to cater traditional foods. The menu was sodium free and included the three sister foods of squash, corn, and beans, along with lean bison meat. The research for the symposium received ethical clearance from the University of Saskatchewan.

## Methods

As an Indigenous community-based health research lab, all research conducted by Morning Star Lodge is community-led and is based on Indigenous Ways of Knowing and Doing. Additionally, the lab has developed respectful and reciprocal long-standing relationships with Indigenous communities across Saskatchewan, including the FHQTC and their member nations. This research emerged out of this relationship and collaboration, the community through their CRAC expressed interest in exploring health and wellness within their communities. Particularly, as it relates to Indigenous older adults' health and well-being. To prevent helicopter research within Indigenous communities, Community-Based Participatory Research (CBPR) design has been found to be effective in equally engaging Indigenous communities in health research where they are held as stakeholders.^5^ CBPR increases the value of research for researchers and the communities being studied.^6,7^ Based on this, Indigenous research methodology and CBPR design informed and framed the research. In addition to these, western quantitative research methods in the form of surveys were adopted to collect data. A CRAC consisting of co-researchers, FHQTC, and ANHN, was formed to guide the entire research process, including survey development and analysis. With the involvement of community members and community health directors from the CRAC, an anonymous survey was distributed at the symposium, where we engaged with 137 co-researchers on various aspects of health including nutrition, exercise, lifestyle, and physical health factors that can contribute to dementia. The co-researchers were from the 11 FHQTC First Nations, the Treaty 4 Territory, and other Indigenous regions in Canada and the United States. Instead of research participants, we use the term co-researchers, where co-researchers do not solely participate in research but rather guide and lead the research. Likewise, the co-researchers guided and led this research process. When adopting western methods in Indigenous research, it is important that the community is involved and determines the methods that are adopted.^8^ As a result, the CRAC was involved in deciding the tools the research adopted to collect data. They also co-developed, reviewed and approved the survey questions, evaluation forms, symposium agenda, keynote speakers, and information tables.

### Data analysis

Data was analyzed using western quantitative methods including softwares such as SPSS and simple excel data analysis in the form of tables. Specifically, descriptive statistics in the form of frequencies and percentages were calculated by access to health services and health education as it relates to nutrition, exercise, and self-management of health issues, as well as holistic health. The results were further analyzed using an Indigenous data analysis method known as Nanâtawihowin cimowina Kika-Môsahkinikêhk Papiskîci-Itascikêwin Astâcikowin (NAKPA). NAKPA data analysis is a process where “a panel of experts, community members, participants, Elders, Knowledge Keepers, and the researchers are gathered together to do the collective data analysis.”^9^ This data analysis approach aligns with the CBPR methodology adopted by directly involving the community in the analysis process. Hence, the data analysis was a collaborative process that involved the community. In this research, the panel consisted of researchers, CRAC members, and FHQTC members. The results were finally reviewed and approved by the CRAC members. All tables and themes presented below were reviewed and approved by the CRAC to ensure the results represented their views. It is important to note that FHQTC members were a part of the entire research process from conception; however, due to unforeseen circumstances, they were unable or unavailable to co-author at this time.

## Results

In this section, we present the results that demonstrate although co-researchers are predisposed to EOD, access to healthcare services remains a major challenge. Further, reiterating the need for health education around nutrition, exercise, self-management of health issues, and holistic health.

### Demographic

Data analysis on the surveys for the environmental scan related to nutrition, exercise, and self-management of health and health issues. A total of 137 surveys were completed and findings emerged a complex overview of the health of Indigenous peoples and access to health services. Overall, 89.1% of the co-researchers were Indigenous. Of this figure, 84% identified as First Nation, 5% identified as Metis, and the remaining identified either as non-Indigenous or did not respond to this question. In particular, 57% of respondents indicated that the nearest health services were at least 60 minutes away. In addition, 48% identified they had at least one morbidity and 24% had more than one morbidity. Sixty-three percent of the respondents identified as female. Overall, the response rates were high, and we recorded 100% response rate on most of the variables; however, on others, we are missing a small percentage of co-researcher responses. See Table 1 below.

**Table 1.** Showing socio-demographic characteristics of the co-researchers.

Co-researchers	Population (%)
First Nations	84
Métis	5.1
Non-Indigenous	10.2
Missing	0.7
Total	100

### Access to health services

**Figure 1.**
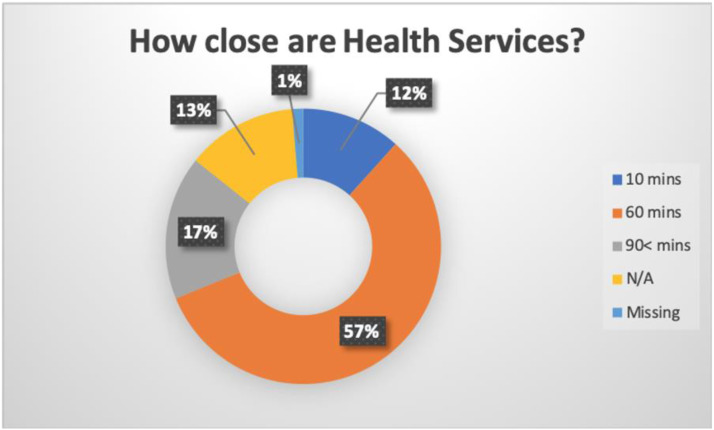
Access to a health facility.

Given the significant cultural roles of most Indigenous older adults, ageing in place is very important for Indigenous families and communities. Despite this, access to healthcare services remains a major challenge for Indigenous older adults, especially those living in remote communities. The results indicate 57% of the co-researchers had to travel approximately 60 minutes to a health facility, while 17% of co-researchers must travel over 90 minutes to the nearest health facility. For older adults living with multiple morbidities, regular access to healthcare is key as ageing predisposes individuals to poorer health outcomes. Figure 1 shows access to the nearest health facility for the co-researchers.

This pie chart demonstrates the distance co-researchers have to travel to access health facilities. The distance ranged from a minimum of 10 minutes to a maximum of over 90 minutes (n = 137). For example, 57% of the co-researchers have to travel about 60 minutes to the closest health facility.

Particularly for this research, half of the co-researchers were living with multiple morbidities, thus making access to healthcare more important for the co-researchers. The findings show that 48% of the co-researchers identified they had at least one morbidity and 24% had more than one morbidity.

Figure 2

**Figure 2.**
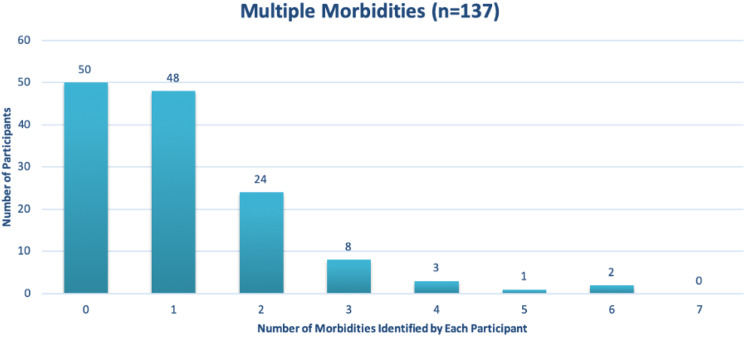
Shows multiple morbidities among co-researchers.

demonstrates the number of co-researchers that self-identify with 0, 1, 2 morbidities. For example, 50 co-researchers identified that they exhibit 0 morbidities and 48 co-researchers identified to have 1 morbidity n = 137.

### Health education

For Indigenous older adults living with multimorbidities in remote communities, health education can support older adults to understand ways nutrition, exercise, and self-management of health issues relate to EOD and their capacity to shape health outcomes. Specifically, health education is needed to help older Indigenous adults understand how multimorbidities relate to their overall health, and how good nutrition, exercise and self-management of health issues can reduce the risk of early onset dementia. Health conditions in the form of elevated blood pressure, elevated cholesterol levels, diabetes mellitus, smoking, depression, obesity, cognitive inactivity, poor diet, poor exercise, and low education have the tendency to cause EOD. Therefore, reducing these health conditions through nutrition, exercise, and the management of health issues can help reduce the risk of EOD. Based on this, the researchers wanted to find out co-researchers' nutritional and exercise patterns as well as their management of health issues.

On nutrition, for example, the results demonstrate most of the co-researchers are not consuming enough vegetables per week, with only a fraction of the co-researchers indicating they consumed vegetables four or more times per week. See Table 2 above.

**Table 2.** Vegetable intake among co-researchers.

Vegetable intake	Co-researchers (n = 137)
0 times a week	9 (6.6%)
1 times a week	31(22.6%)
2 times a week	35 (25.5%)
3 times a week	29 (21.2%)
4 times a week	4 (2.9%)
5 times a week	4 (2.9%)
6 times a week	0
7 times a week	0
Missing	25 (18.3%)

However, a substantial number of the co-researchers use tobacco with as high as 59 (43.1%) of the co-researchers using tobacco more than five times a week. In addition to nutrition, lifestyle modification is key to reducing the risk of EOD. See Table 3 below.

**Table 3.** Tobacco use among co-researchers.

Tobacco	Co-researchers (n = 137)
0 times a week	55 (40.1%)
1 times a week	4 (2.9%)
2 times a week	4 (2.9%)
3 times a week	6 (4.4%)
4 times a week	9 (6.6%)
5+ times a week	59 (43.1%)

But unlike tobacco, the results show most of the co-researchers did not consume alcohol with as high as 95 (69.3%) of the co-researchers reporting they do not consume alcohol. See Table 4 below.

**Table 4.** Alcohol use among co-researchers.

Alcohol use	Co-researcher (n = 137)
0 times a week	95 (69.3%)
1 times a week	23 (16.8%)
2 times a week	10 (7.3%)
3 times a week	5 (3.6%)
4 times a week	2 (1.5%)
5+ times a week	1 (.7%)
Missing	1 (.7%)

### Holistic health

Importantly, our study also found the need for health education to adopt a holistic approach to health including physical, spiritual, emotional, and mental health. The co-researchers indicated the importance for health education to capture all these multi-dimensions of health. A holistic approach would ensure that physical, mental, spiritual, and emotional healthcare are regarded as equally as important. For example, mental health can influence lifestyle choices such as tobacco use. A balance among these multi-dimensions of health is therefore needed to enhance well-being. Education around nutrition, exercise, and management of health issues must therefore be promoted from the prism of holistic health.

Figure 3

**Figure 3.**
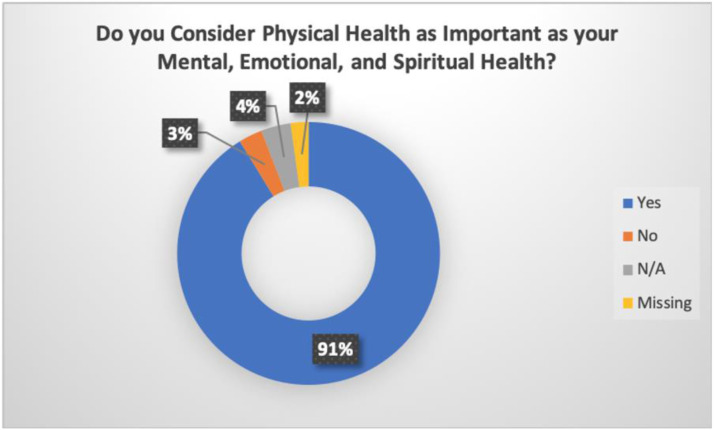
Holistic health.

demonstrates the holistic dimensions of health important to co-researchers (n = 137). Ninety-one percent of the co-researchers considered physical health to be as important as mental, emotional, and spiritual health.

## Discussion

Canada’s history of colonialism and racism accounts for the health inequities that Indigenous people face today.^10,11,12^ Particularly for Indigenous older adults, colonialism and racism have been found to be impediments to healthy ageing in place.^13^ These continue to serve as barriers limiting Indigenous older adults' access to healthcare.^14^ It has been observed that even research on healthy ageing in place often ignores older Indigenous peoples’ voices and experiences.^13,11^ Based on this, most of the barriers to health experienced by the Indigenous older adults in this study, including access to healthcare, ageing technology, and health education, are also linked to colonialism. Therefore, it is suggested that colonialism and racism be addressed in order to enhance Indigenous older adults' access to healthcare including technology.^1^

For this research, it is imperative to examine barriers of access to healthcare caused by infrastructure and cultural appropriateness. Older adults living in remote communities face a heightened risk of dementia,^15^ resulting from multimorbidities. Access to healthcare services remains a major challenge for Indigenous older adults living in remote communities in Canada.^1,2^ As ageing comes with health complications, regular access to healthcare services including dementia care and other specialized services are necessary to enhance the health and well-being of older adults. Particularly for Indigenous older adults, Jones et al. observed “[a]ging within Indigenous communities interacts with social inequities; consequently, older Indigenous adults may be more likely to require regular and specialized healthcare.”^1^ Indigenous older adults are predisposed to multimorbidities that heighten the risk of EOD. The study found that most of the co-researchers had multimorbidities that may heighten the risk of EOD. Meanwhile, access to healthcare services was a challenge for the majority of the co-researchers who had to travel between 60 and 90 minutes to the nearest health facility. The results from this study corroborate previous studies suggesting that access to healthcare services remains a challenge for remote Indigenous communities in Canada.^15^ Limited access to dementia services and specialists in remote communities prevents older adults from ageing in place and further exacerbates the burden of caring for family members living with dementia.^15^ Additionally, the findings from this research also support previous studies that have found high rates of multimorbidities among Indigenous populations.^16,17^ It is important to also note that the presence of multimorbidity among co-researchers and the inaccessibility of healthcare services are directly linked to the continuous impact of colonialism.^18,19^

Although limited access to healthcare is a major barrier to ageing within remote Indigenous communities, technology can support older adults’ capacity to age in place by monitoring their health data and health status. Especially for Indigenous older adults living with multimorbidities that predispose them to the risk of EOD, technology can enhance care and access to health services. Therefore, the findings further highlight the importance of technology to enhance healthy ageing among Indigenous communities. As Bourassa et al. observed, the introduction of ageing technology can enhance and augment dementia care.^15^

Technology coupled with health education supports the monitoring of health data and health status as well as promotes dementia education, which can help delay or prevent EOD. Based on this, the research sought to engage co-researchers in a dialogue on nutrition, exercise, and self-management of health and the relationship to cognitive function and dementia. Fruits and vegetables, for example, play a key role in ageing-related disease prevention.^20^ On nutrition, the research wanted to determine tobacco use, alcohol consumption, and vegetable intake among co-researchers. It emerged that, contrary to previous research that found higher alcohol use among Indigenous communities,^21^ the results showed lower alcohol consumption among co-researchers with as high as 69.3% of co-researchers indicating zero alcohol consumption within a week. Similarly, the research found that vegetable intake was relatively low among the co-researchers further confirming previous studies, showing lower vegetable intake among Indigenous populations.^22^ Given that the workshop took place in September, which happens to be the gardening season in Saskatchewan, one would have expected that vegetable consumption among the co-researchers would be much higher. Community gardening has been found to increase vegetable intake.^23^ Additionally, the results indicated high tobacco use among the co-researchers thus confirming the studies by Minichiello et al. that show high tobacco use among Indigenous communities.^24^ Given that most of the co-researchers were older adults, smoking increases the risk of developing dementia.^25^ Traditionally, tobacco is sacred and plays a very important role in most Indigenous cultures but studies show an increase in recreational tobacco use among Indigenous communities.^26^ Again, colonialism has been found to be the major cause of high tobacco use among Indigenous communities.^27^

Access to culturally appropriate technology can help monitor health information,^2^ including sugar levels, blood pressure, and weight. Health information technology has been found to support older adults in self-monitoring of health status.^28^ This can further support health education which forms an important component of this research. Using new technology to monitor health data brings awareness about how diet, exercise, and lifestyle can contribute to the prevention of EOD. Furthermore, informing co-researchers on effective health management using technologies has led to an authentic engagement on the ways in which health management can contribute to community well-being.

Indigenous communities view health holistically. The study found that good health to the co-researchers means maintaining a balance among all the dimensions of health including physical, spiritual, emotional, and mental health. Thus, corroborating early findings by Graham and Stamler that found similar themes of physical, spiritual, emotional, and mental health as being important to Indigenous communities.^29^ Our study found that health education on nutrition, exercise and the management of health conditions must be addressed through these themes. Additionally, cultural understanding of dementia is very important to addressing the conditions that heighten the risk of EOD among Indigenous communities. Indigenous communities view dementia differently from mainstream Western Canadian society. As Jacklin and Walker observed, Indigenous peoples view dementia as a natural part of the life cycle.^30^ Framing health education on dementia around these cultural lenses is key to enhancing the health and well-being of Indigenous older adults.

Access to health service is an important social determinant of health for Indigenous peoples in Canada.^31^ The research shows a gap and emphasizes the need for technology including telehealth and other accessible forms of healthcare services for remote Indigenous communities. Additionally, the findings highlight the areas of concern that healthcare professionals working within Indigenous communities need to focus on in a culturally safe way. Particularly, on issues surrounding nutrition, exercise and the management of health conditions, we provide baseline information to inform decision making among healthcare professionals working within Indigenous communities. For example, vegetable intake, alcohol, and tobacco use are issues that demand a holistic approach to tackle within Indigenous communities. The information provided by this study is important for healthcare professionals working within Indigenous communities to adopt a culturally safe approach to working with Indigenous peoples. Furthermore, this study could inform future research and initiatives investigating the accessibility to nutritious foods by means of a culturally safe and relevant approach. Again, the study shows the importance of health education that is collaborative with community members. This kind of collaborative work is necessary to help build respectful and trustful relationships that can enhance the health and well-being of Indigenous communities.

## Conclusion

The purpose of the symposium was to assess Indigenous older adults’ accessibility to and readiness for new technologies to support in the monitoring of their health data and health status, as well as dementia education. The results show that access to healthcare services remains a challenge for most remote Indigenous communities. Particularly for Indigenous older adults living with multimorbidities, regular access to healthcare is key to healthy ageing in place. It has emerged that technology combined with health education can support Indigenous older adults’ capacity to age healthily in place. First Nations communities are requesting more information that holds relevant research around Indigenous health, so that they may have appropriate information to learn from as well as hold overall control over their health outcomes. These findings successfully informed a follow-up AGE-WELL project exploring technologies with Indigenous older adults to monitor health status and promote healthy behaviours and lifestyles, with the goal of enhancing the health and well-being of Indigenous communities. The follow-up project employs health tools and education to support Indigenous older adults to understand the ways nutrition, exercise, multiple morbidities, and self-management of health issues relate to early-onset dementia and their capacity to shape health outcomes.
